# Single-Center Experience with Sacubitril/Valsartan in Patients with Congenital Heart Disease

**DOI:** 10.1007/s00246-025-03938-x

**Published:** 2025-07-04

**Authors:** Anusha Konduri, Ashley Duimstra, Ray Lowery, Sunkyung Yu, Ashley Huebschman, Bronwyn Crandall, Tiffany Hunter, Heang M. Lim, Amanda D. McCormick, Kurt R. Schumacher, David M. Peng

**Affiliations:** University of Michigan Congenital Heart Center, Ann Arbor, MI USA

**Keywords:** Congenital heart disease, Heart failure, ARNI

## Abstract

Heart failure is a significant cause of morbidity and mortality in patients with congenital heart disease (CHD). While clinical guidelines for acquired cardiovascular diseases exist, evidence-based therapies for heart failure in CHD are lacking. Angiotensin II receptor blocker and neprilysin inhibitor (ARNI) therapy has shown efficacy in adults with heart failure, reducing cardiovascular mortality and hospitalizations, but its use in pediatric CHD patients remains underexplored. This study aimed to evaluate the safety and efficacy of Sacubitril/Valsartan (ARNI) in pediatric and young adult patients with CHD. We conducted a retrospective chart review of 29 patients who received ARNI therapy between August 2021 and December 2023. The patients’ age ranged from 5.3 months to 22.8 years. Fifteen of the 29 patients (52%) had single ventricle CHD. The median time for follow-up since ARNI initiation was 8 months (range 6 days–2.4 years). Twelve (41%) patients experienced hypotension which necessitated dose adjustments, temporary withholding of the medication, or discontinuation. Additionally, 2 patients developed acute kidney injury. The medication had to be discontinued in 7 patients (24%) due to hypotension (3 or 10%), AKI (2 or 7%) and progression of heart failure needing advanced cardiac therapies (2 or 7%). A comparative analysis of clinical and laboratory data before and after ARNI therapy revealed a significant reduction in systolic blood pressure (*p* = 0.01), as well as increases in serum creatinine and potassium levels (*p* = 0.02 and *p* = 0.03, respectively). Additionally, there was a trend toward improvement in ventricular systolic function observed on echocardiogram after ARNI therapy; however, this was in the context of patients receiving concomitant other oral heart failure medications. Our findings highlight the need for careful monitoring and individualized management of ARNI therapy in pediatric CHD patients. Larger, well-designed trials are essential to establish clear treatment guidelines and optimize the use of ARNI therapy in this population.

## Background

Heart failure is a significant cause of morbidity and mortality in patients with congenital heart disease (CHD), affecting both children and adults with a variety of underlying cardiac anomalies [1]. These patients may develop heart failure with reduced ejection fraction (HFrEF) or preserved ejection fraction (HFpEF), and the management of heart failure in CHD presents unique challenges due to the complexity of congenital defects [2]. While there are well-established clinical guidelines for heart failure management in adults with acquired cardiovascular diseases, there is a lack of evidence-based therapies and specific guidelines tailored to patients with CHD. Pediatric heart failure treatment often relies on data extrapolated from adult studies, and the absence of pediatric-specific clinical trials further complicates the development of optimal therapeutic strategies.

The PANORAMA-HF trial represents one of the few large-scale efforts to evaluate pharmacologic therapy specifically in pediatric heart failure [3]. This study found no significant difference in clinical outcomes between sacubitril/valsartan and enalapril in children with systemic left ventricular dysfunction, although both treatments were associated with clinically meaningful improvements. Notably, patients with single ventricle physiology and systemic right ventricles were excluded, despite the high prevalence of heart failure in these subgroups. This underscores the need for additional research to assess the safety and efficacy of novel therapies in broader CHD populations. Newer pharmacologic interventions, such as angiotensin receptor blocker–neprilysin inhibitors (ARNI), have become central to guideline-directed medical therapy (GDMT) for heart failure in adults. According to guidelines from the American College of Cardiology (ACC), American Heart Association (AHA), and Heart Failure Society of America (HFSA), ARNI are given a Class I recommendation for the treatment of HFrEF [4].

Sacubitril/valsartan, the only FDA-approved ARNI for heart failure, combines valsartan (an angiotensin II receptor blocker) and sacubitril (a neprilysin inhibitor). Valsartan works by reducing vasoconstriction, sodium retention, and cardiac remodeling, while sacubitril enhances natriuretic peptide activity, promoting vasodilation, diuresis, and improved fluid balance [5, 6]. Clinical trials in adults without CHD, including PARADIGM-HF, have demonstrated that sacubitril/valsartan significantly reduces the risk of death and hospitalization in patients with HFrEF [7]. The PARAGON-HF trial, although not meeting its primary endpoint in HFpEF, suggested potential benefits for patients with lower ejection fractions and elevated natriuretic peptide levels [8]. Despite these promising findings in adults, data on ARNI use in pediatric heart failure—especially in children with CHD—remain limited. The exclusion criteria and outcomes of PANORAMA-HF highlight important gaps in our understanding of how these therapies perform in complex congenital populations, pointing to the critical need for future investigation.

### Aim and Objectives

The aim of this study was to evaluate the safety, efficacy, and clinical outcomes of sacubitril/valsartan (ARNI) therapy in pediatric patients with CHD. Specifically, the study sought to assess the safety of ARNI therapy in this population, evaluate its efficacy by examining changes in New York Heart Association (NYHA) class, hospitalizations due to cardiac etiology, and systolic ventricular function on echocardiogram, and analyze laboratory data before and after therapy to assess its effect on serum electrolytes and renal function in patients with CHD.

## Methodology

### Study Type

This study was a single-center, retrospective cohort study conducted from August 1, 2021, to December 31, 2023. Institutional Review Board (IRB) approval was obtained prior to data collection.

### Patients and ARNI Therapy

Patients included in the study were those diagnosed with CHD who received ARNI therapy during the study period. Patients with heart failure and moderately depressed systolic function or worse on echocardiogram were categorized as HFrEF and patients with heart failure symptoms with normal or mildly depressed systolic function were categorized as HFpEF. Patient demographics, underlying diagnoses, clinical status, and NYHA class were recorded. In accordance with the ACTION (Advanced Cardiac Therapies Improving Outcomes Network) dosing recommendations, it was observed that Sacubitril/Valsartan therapy at our center was generally initiated at a starting dose of 0.8 mg/kg/dose twice daily for patients weighing < 50 kg, with gradual titration to a target dose of 3.1 mg/kg/dose twice daily. For patients weighing ≥ 50 kg, the starting dose was 24–26 mg/dose twice daily, with titration to a maximum dose of 97–103 mg/dose twice daily [9]. However, the dosing and up-titration were not always strictly aligned with the ACTION recommendations; in some cases, therapy was initiated at a lower dose, and up-titration was done more slowly based on the patient's clinical status. Patients previously on angiotensin converting enzyme inhibitors (ACEi) or angiotensin receptor blockers (ARBs) had these medications discontinued and were transitioned to ARNI therapy 36–48 h later.

### Data and Outcomes

Patient data were collected from the electronic medical record, including baseline and follow-up clinical information, echocardiographic results, and laboratory values. Outcomes evaluated included:

- Safety outcomes, including blood pressure, incidence of hypotension, Acute Kidney Injury (AKI), hyperkalemia and angioedema
- Efficacy outcomes, including NYHA class, hospitalizations due to cardiac etiology and qualitative systolic function on echocardiogram. Ventricular function was assessed qualitatively using echocardiogram reports obtained through retrospective chart review.
- Laboratory data including serum creatinine, blood urea nitrogen (BUN), estimated glomerular filtration rate (eGFR), serum potassium, and natriuretic peptide levels
- Other therapies used in conjunction with ARNI

### Analysis

Data were summarized as median with interquartile range, or mean ± standard deviation (SD) for continuous variables, and frequency (percentage) for categorical variables. Pre- and post-ARNI initiation data were compared using paired t-tests for continuous variables and McNemar’s test for categorical variables. A *p*-value of < 0.05 was considered statistically significant. Due to the relatively small sample size, Cohen’s d effect size was also calculated to assess the magnitude of change in continuous variables, with thresholds for small (0.2), medium (0.5), and large (0.8) effects [10]. All statistical analyses were performed using SAS version 9.4 (SAS Institute, Cary, NC, USA).

## Results

### Baseline Characteristics

Our analysis included 29 patients with CHD who had received ARNI during the study period. Patient age ranged from 5.3 months to 22.8 years. Fifteen of the 29 patients (52%) had single ventricle CHD (Table 1). The most common indication for starting the medication was HFrEF (n = 20, 69%) (Table 2). Six patients had HFpEF including 2 patients with Fontan associated protein losing enteropathy (PLE). At initiation, most of these patients were already on other heart failure medications such as diuretics, beta-blockers, ACEi, sodium glucose cotransporter 2 inhibitors (SGLT2i), and mineralocorticoid receptor antagonists (MRA). ARNI was initiated in 17 (58.6%) patients as inpatients and 12 (41.4%) as outpatients. At the time of ARNI initiation, 45% of patients (13 out of 29) were transitioned from ACEi/ARB and 24% of patients (7 out of 29) were transitioned from intravenous milrinone. The median time for follow-up since ARNI initiation was 8 months (range 6 days–2.4 years) (Table 3).

**Table 1:** Demographics and clinical encounter characteristics prior to ARNI initiation (N = 29)

Male sex	21 (72.4)
Caucasian race	24 (82.8)
Hispanic ethnicity	0 (0.0)
Primary cardiac diagnosis	
HLHS	11 (37.9)
DORV	4 (13.8)
Unbalanced AVSD	2 (6.9)
Complete AVSD	1 (3.4)
TOF/AVSD	1 (3.4)
ccTGA	1 (3.4)
VSD	1 (3.4)
Shone’s complex	1 (3.4)
PA/VSD	3 (10.3)
TAPVR	1 (3.4)
Aortic arch hypoplasia	1 (3.4)
Aortic insufficiency	1 (3.4)
Dilated aortic root	1 (3.4)
Single ventricle	15 (51.7)
Systemic ventricle	
Left	0/15 (0.0)
Right	15/15 (100.0)
Age at clinical encounter prior to ARNI initiation, years	13.4 (2.7–19.5)
Weight at clinical encounter prior to ARNI initiation, kg	42.2 ± 30.6
Height at clinical encounter prior to ARNI initiation, cm	131 ± 43.3
BSA at clinical encounter prior to ARNI initiation, m^2^	1.2 ± 0.64
BMI at clinical encounter prior to ARNI initiation, kg/m^2^	20.2 ± 6.3
NYHA class at clinical encounter prior to ARNI initiation	
I	4 (13.8)
II	6 (20.7)
III	6 (20.7)
IV	1 (3.4)
Unknown	12 (41.4)
Systolic BP at clinical encounter prior to ARNI initiation, mmHg (N = 28)	111 ± 16.3
Systolic BP percentile for patients age > 2 and < 18 years (N = 13)	74.3 (34.5–97.9)
Systolic BP z-score for patients age > 2 and < 18 years (N = 13)	1.2 ± 2.0
Systolic BP for patients age > 18 years, mmHg (N = 8)	113 ± 17.0
Diastolic BP at clinical encounter prior to ARNI initiation, mmHg (N = 27)	61.9 ± 9.4
Diastolic BP percentile for patients age > 2 and < 18 years (N = 12)	56.7 (28.2–90.0)
Diastolic BP z-score for patients age > 2 and < 18 years (N = 12)	0.45 ± 1.2
Diastolic BP for patients age > 18 years, mmHg (N = 8)	64.9 ± 7.4
Hospitalization due to heart failure within one year prior to ARNI initiation	11 (37.9)

*Data are presented as N (%) for categorical variables and mean ± standard deviation or median (interquartile range) for continuous variables

**Table 2:** Clinical Characteristics at ARNI Initiation (N = 29)

Age at ARNI initiation, years	13.4 (2.7–19.5)
Weight at ARNI initiation, kg (N = 25)	41.3 ± 31.2
Height at ARNI initiation, cm (N = 25)	129 ± 44.1
BSA at ARNI initiation, m^2^ (N = 25)	1.2 ± 0.64
BMI at ARNI initiation, kg/m^2^ (N = 25)	20.2 ± 6.6
Indication for ARNI	
HFrEF	20 (69.0)
HFpEF	5 (17.2)
HFpEF and PLE	1 (3.4)
PLE	1 (3.4)
RV dysfunction and TR	1 (3.4)
Hypertension	1 (3.4)
Initial ARNI dose, mg	50.0 (16.8–50.0)
Initial ARNI dose for patients < 50 kg, mg/kg (N = 18)	0.95 (0.79–1.6)
Initial ARNI dose for patients > 50 kg, kg (N = 11)	50 (50–50)
Final ARNI dose, mg	100 (34.0–200)
Final ARNI dose for patients < 50 kg, mg/kg (N = 18)	1.6 (1.1–2.7)
Final ARNI dose for patients > 50 kg, kg (N = 11)	100 (50–100)
ARNI initiated inpatient	17 (58.6)
Other heart failure medications at time of ARNI initiation	29 (100.0)
ACE inhibitor/ARB	13 (44.8)
IV Milrinone	7 (24.1)
ARB	0 (0.0)
Beta blocker	15 (51.7)
Diuretic	26 (89.7)
MRA	20 (69.0)
PDE5 inhibitor	11 (37.9)
SGLT2 inhibitor	12 (41.4)

*Data are presented as N (%) for categorical variables and mean ± standard deviation or median (interquartile range) for continuous variables

**Table 3:** Characteristics at most recent clinical encounter (N = 29)

Age at most recent clinical encounter, years (N = 27)	15.0 (3.6–21.4)
Time from ARNI initiation to most recent clinical encounter, months (N = 27)	8.0 (4.9–18.2)
Weight at most recent clinical encounter, kg (N = 27)	48.3 ± 30.7
Height at most recent clinical encounter, cm (N = 27)	141 ± 38.6
BSA at most recent clinical encounter, m^2^ (N = 27)	1.3 ± 0.60
BMI at most recent clinical encounter, kg/m^2^ (N = 27)	21.2 ± 6.7
NYHA class at most recent clinical encounter	
I	6 (20.7)
II	7 (24.1)
III	4 (13.8)
IV	3 (10.3)
Unknown	9 (31.0)
Systolic BP at most recent clinical encounter, mmHg (N = 26)	101 ± 16.7
Systolic BP percentile for patients age > 2 and < 18 years (N = 14)	36.0 (5.7–64.7)
Systolic BP z-score for patients age > 2 and < 18 years (N = 14)	− 0.49 ± 1.4
Systolic BP for patients age > 18 years, mmHg (N = 8)	108 ± 18.7
Diastolic BP at most recent clinical encounter, mmHg (N = 24)	57.2 ± 11.4
Diastolic BP percentile for patients age > 2 and < 18 years (N = 13)	45.8 (14.0–72.5)
Diastolic BP z-score for patients age > 2 and < 18 years (N = 13)	− 0.35 ± 1.0
Diastolic BP for patients age > 18 years, mmHg (N = 8)	62.8 ± 13.3
ARNI discontinued	7 (24.1)
Reason for discontinuation	
Hypotension	2/7 (28.6)
AKI	2/7 (28.6)
Heart failure exacerbation and started IV inotropes	1/7 (14.3)
VAD placement	1/7 (14.3)
Cardiac arrest, likely secondary to hypotension and compromising to coronary perfusion	1/7 (14.3)
Adverse events	13 (44.8)
AKI	2 (6.9)
Hypotension	12 (41.4)
Angioedema	0 (0.0)
Hyperkalemia	0 (0.0)
Hospitalization due to heart failure within one year post-ARNI initiation (N = 27)	5 (18.5)
Other heart failure medications at most recent clinical encounter (N = 27)	26 (96.3)
ACE inhibitor	1 (3.7)
ARB	0 (0.0)
Beta blocker	15 (55.6)
Diuretic	23 (85.2)
MRA	21 (77.8)
PDE5 inhibitor	9 (33.3)
SGLT2 inhibitor	20 (74.1)
Underwent heart transplant	4 (13.8)
Deceased	5 (17.2)
Time from ARNI initiation to death, months (N = 5)	3.0 (2.1–5.1)

*Data are presented as N (%) for categorical variables and mean ± standard deviation or median (interquartile range) for continuous variables

### Safety Outcomes and Adverse Events

A comparative analysis of blood pressure before and after ARNI therapy revealed a significant reduction in systolic blood pressure (mean 112 ± SD 15.9 vs 102 ± 16.2, *p* = 0.01) (Table 4). Twelve (41%) patients experienced hypotension which necessitated dose adjustments, temporary withholding of the medication, or discontinuation (Table 3). Time from ARNI initiation to hypotension had a median (IQR) of 9.5 (1.0–175) days, ranging from day 0 to 1.1 years. The assessment of hypotension was based on clinical judgment and individual patient circumstances, rather than strictly on predefined blood pressure thresholds. Additionally, two patients discontinued the medication due to concerns for AKI, occurring at 15 and 88 days after initiation, respectively. However, both events took place in the context of other concomitant medication adjustments and during hospitalizations for heart failure exacerbations. None of our patients experienced angioedema or clinically significant hyperkalemia. The medication had to be discontinued in 7 patients (24%) due to hypotension (n = 3), AKI (n = 2), or progression of heart failure needing advanced cardiac therapies (n = 2). Time to discontinuation for side effects ranged from 1 to 88 days. Only one patient had a dose reduction prior to discontinuation. For the others, dose reduction was not attempted because they were already on low doses at the time adverse effects occurred. The timeline of dose adjustments varied widely from 2 days (inpatients) to several months (outpatients). Of the 29 patients, 10 successfully reached the target dose, 2 patients required dose reduction due to adverse effects, and 8 maintained the target dose throughout the study period. Many patients could not be up-titrated due to side effects including hypotension.

**Table 4:** Changes in clinical characteristics from pre-ARNI initiation to most recent clinical encounter (N = 27)

	Pre-ARNI encounter	Most recent encounter	Change (most recent – pre)	*p*^§^	Effect Size *d*
NYHA class at clinical encounter					
I	4 (14.8)	6 (22.2)			
II	6 (22.2)	7 (25.9)			
III	6 (22.2)	4 (14.8)			
IV	1 (3.7)	3 (11.1)			
Unknown	10 (37.0)	7 (25.9)			
Systolic BP at clinical encounter, mmHg (N = 25)	112 ± 15.9	102 ± 16.2	− 10.1 ± 19.0	**0.01**	**0.53**
Systolic BP percentile at clinical encounter (N = 13)	74.3 (34.5–97.9)	37.1 (13.4–64.7)	− 28.8 (− 42.3–1.8)		
Systolic BP z-score at clinical encounter (N = 13)	1.2 ± 2.0	− 0.33 ± 1.3	− 1.6 ± 1.9		0.81
Systolic BP for patients age > 18 years, mmHg (N = 8)	113 ± 17.0	108 ± 18.7	− 5.3 ± 21.6		
Diastolic BP at clinical encounter, mmHg (N = 22)	62.4 ± 9.3	58.5 ± 10.7	− 3.8 ± 13.4	0.19	0.29
Diastolic BP percentile at clinical encounter (N = 11)	55.7 (26.4–88.2)	45.8 (14.0–73.0)	− 13.0 (− 38.5–0.90)		
Diastolic BP z-score at clinical encounter (N = 11)	0.37 ± 1.3	− 0.25 ± 0.92	− 0.62 ± 1.2		0.53
Diastolic BP for patients age > 18 years, mmHg (N = 8)	64.9 ± 7.4	62.8 ± 13.3	− 2.1 ± 16.6		
	Within 1 Yr. pre-ARNI	Within 1 Yr. Post-ARNI		*p*^§^	
Hospitalization due to heart failure	10 (37.0)	5 (18.5)		0.13	

*Data are presented as N (%) for categorical variables and mean ± standard deviation or median (interquartile range) for continuous variables

^§^*p*-value from McNemar’s test for categorical variables and paired t-test for continuous variables

*Cohen's *d* effect size was calculated as mean change between the two time points divided by the pooled standard deviation of the two time points, indicating small (.20), medium (.50), and large (.80)

One patient, a 10-month-old with a double outlet right ventricle/hypoplastic left ventricle, palliated with a modified Blalock-Thomas-Taussig shunt, admitted to the hospital with HFpEF, experienced a cardiac arrest after receiving one dose of sacubitril/valsartan.

### Laboratory Data

A comparative analysis of laboratory data before and after ARNI therapy revealed an increase in serum creatinine (0.69 ± 0.41vs 0.81 ± 0.55, *p* = 0.02) and potassium levels (4.0 ± 0.70 vs 4.5 ± 0.55, *p* = 0.03) (Table 5). In our cohort, although we observed a trend toward increased BNP levels after initiation, our sample size and short follow-up duration limited our ability to perform meaningful statistical analysis.

**Table 5:** Changes in lab characteristics from pre-ARNI initiation to most recent clinical encounter (N = 23)

Labs	Pre-ARNI encounter	Most recent encounter	Change (most recent – pre)	*p*^§^	Effect size *d*
BUN, mg/dL	19.0 ± 6.4	24.5 ± 12.2	5.4 ± 12.9	0.06	0.42
Creatinine, mg/dL	0.69 ± 0.41	0.81 ± 0.55	0.12 ± 0.23	**0.02**	**0.54**
eGFR^†^, mL/min/1.73m^2^	99.2 ± 32.5	91.0 ± 29.9	− 8.1 ± 21.2	0.08	0.38
Potassium, mmol/L	4.0 ± 0.70	4.5 ± 0.55	0.43 ± 0.87	**0.03**	**0.49**
BNP, pg/mL (N = 10)	292 ± 239	878 ± 1,458	586 ± 1,448		0.40
NT-proBNP, pg/mL (N = 5)	2,257 ± 1,639	10,251 ± 14,874	7,994 ± 14,374		0.56

*Data are presented as mean ± standard deviation

^§^*p*-value from paired t-test

*Cohen's *d* effect size was calculated as mean change between the two time points divided by the pooled standard deviation of the two time points, indicating small (.20), medium (.50), and large (.80)

^†^Using ‘Bedside Schwartz’ formula for Age < 18 years and CKD-EPI 2021 equation for age ≥ 18 years

### Efficacy Outcomes

**Fig. 1:**
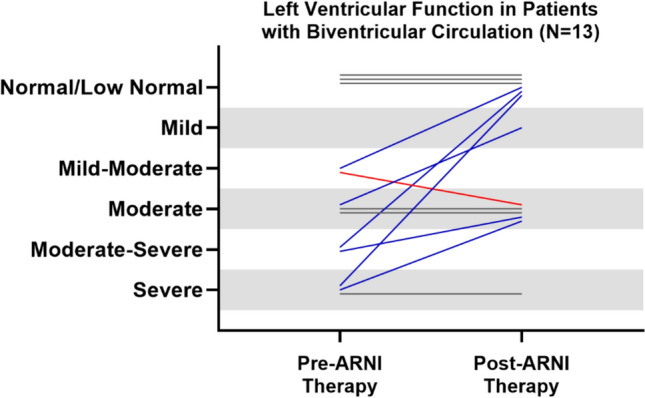
Changes in qualitative assessment in LV function in patients with biventricular circulation

**Fig. 2:**
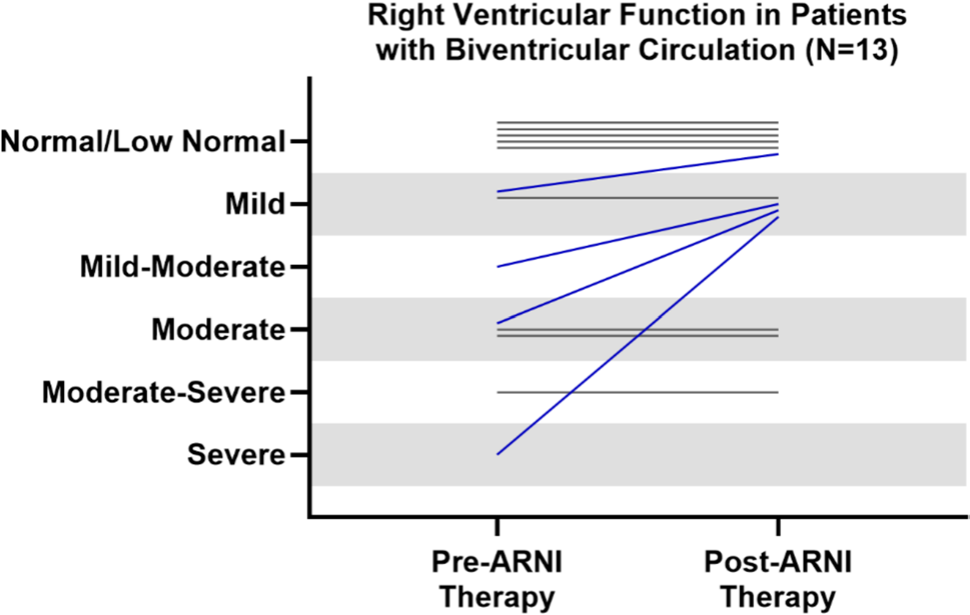
Changes in qualitative assessment in RV function in patients with biventricular circulation

Some patients showed an improvement in the qualitative assessment of ventricular function in both the left and right ventricles among those with biventricular circulation, while others did not exhibit any significant changes (Figs. 1 and 2). However, due to the limited sample size, statistical analysis could not be performed. The improvement occurred in the context of concomitant use of other oral heart failure medications. We noted a trend towards improvement in hospitalizations before and after ARNI therapy (from 37 to 19%), although this did not reach a statistical significance (*p* = 0.13).

### Single Ventricle CHD

**Fig. 3:**
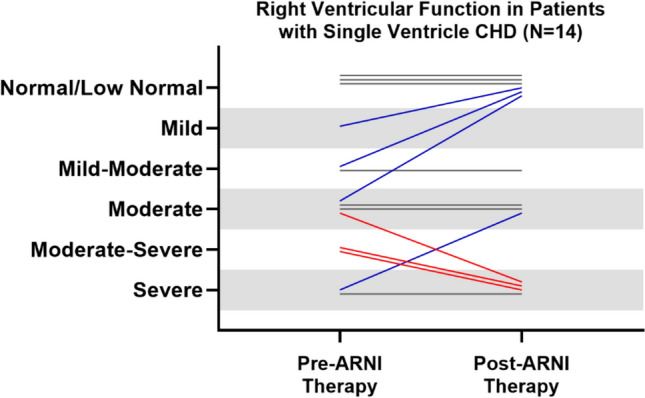
Changes in qualitative assessment in systemic ventricular function in patients with single ventricle CHD

All 15 patients with single ventricle CHD in our cohort had a systemic right ventricle. At the time of starting ARNI, 3 patients were at Stage 1 palliation, 1 was at Stage 2 palliation, and 11 had completed the Fontan procedure. Hypotension was observed in 9 out of the 15 patients (60%), and 1 patient developed AKI. The medication had to be discontinued in 4 patients (27%) due to hypotension (n = 2), AKI (n = 1), or progression of heart failure needing advanced cardiac therapies (n = 1). Some patients showed an improvement in qualitative assessment of systolic function of the systemic ventricle on echocardiogram (Fig. 3). However, the results were mixed, with some patients showing no change and others experiencing a deterioration in ventricular function, making it challenging to directly attribute these changes to ARNI therapy. Additionally, due to the limited sample size, statistical analysis was not feasible. It is important to note that these changes occurred in the context of concomitant oral heart failure medications.

## Discussion

Our study provides important insights into the use of sacubitril/valsartan in patients with CHD, particularly those with single ventricle physiology, a group often excluded from prior clinical trials. Despite the widespread use of ARNI in adults, its use in pediatric patients with congenital defects has been less studied. Consequently, clinical guidelines for pediatric CHD patients are still evolving, with much of the current treatment based on extrapolated data from adult populations [11]. With U.S. Food and Drug Administration approval of sacubitril/valsartan, ARNI will likely continue to be prescribed for pediatric heart failure. Our findings contribute to the growing, yet limited, body of literature on the use of ARNI in pediatric heart failure, adding valuable data for this inadequately studied group.

A key finding of our study was the high incidence of hypotension, observed in 41% of patients, which necessitated dose adjustments or discontinuation in several cases. This is consistent with previous reports indicating an increased risk of hypotension with ARNI [12, 13]. The rates of hypotension in our study were higher than other reported studies with pediatric patients suggesting that the CHD population is more prone to hypotension as compared to non-CHD patients. Our findings underscore the importance of vigilant blood pressure monitoring, especially given the risk of exacerbating low cardiac output. One of our patients, a 10-month-old with a double outlet right ventricle/hypoplastic left ventricle, palliated with a modified Blalock-Thomas-Taussig shunt, experienced a cardiac arrest after receiving 1 dose of sacubitril/valsartan from ventricular tachycardia, potentially triggered by hypotension caused by ARNI, which may have led to coronary ischemia due to diastolic steal in a shunted patient.

While hypotension was the most common adverse event, we also observed two cases of AKI, further emphasizing the need for careful renal monitoring when using ARNI in this population. However, none of our patients experienced angioedema or clinically significant hyperkalemia, which are known adverse effects of ARNI therapy in adults [7].

In our cohort, we observed a trend toward increased BNP levels following initiation of sacubitril/valsartan. Although our sample size and follow-up duration were insufficient for statistical analysis, this finding aligns with prior reports, including the PARADIGM-HF trial, which demonstrated that BNP may initially rise due to neprilysin inhibition [14].

We observed an improvement in the ventricular systolic function in some of our patients, as well as a trend toward reduction in hospitalizations, among CHD patients receiving ARNI therapy. While these findings are promising, the small sample size and retrospective nature of our study require cautious interpretation. These results underscore the complexities of managing heart failure in CHD, especially in pediatric populations where evidence on newer pharmacologic therapies, such as ARNI, remains limited. Studies in adults with CHD have shown mixed results regarding the efficacy of ARNI [15]. Some small studies and case series, have demonstrated improvements in biomarkers, RV function, NYHA status, and quality of life in adult congenital heart disease (ACHD) patients, suggesting that ARNI could have a beneficial role in this population [16–19]. However, Maurer et al. and Yan et al. reported limited or no significant improvements in ACHD patients with ARNI use [13, 20]. The mixed results reflect the heterogeneity of CHD and highlight the need for individualized approaches to treatment.

The decision to discontinue ARNI in 24% of our cohort, particularly due to hypotension and AKI, raises important questions about how best to balance the potential therapeutic benefits of ARNI with the risks in this vulnerable patient group. Although ARNI has proven beneficial in improving outcomes for adults with heart failure, its use in pediatric CHD patients remains fraught with challenges due to the variability in response and the risk of serious adverse events. Given the high incidence of adverse events in our cohort, we suggest that a more tailored approach to dosing and patient monitoring may be necessary for this population. It is possible that initiating treatment with lower doses and gradually titrating upward may help reduce the risk of adverse events while still providing therapeutic benefits. Additionally, more rigorous and frequent monitoring protocols particularly for blood pressure and renal function should be considered in pediatric CHD patients receiving ARNI therapy to mitigate potential risks.

In our single-ventricle patient population, a significant proportion experienced hypotension with sacubitril/valsartan. Despite this, we observed an improvement in systemic ventricular systolic function in some of our patients. This finding is noteworthy, as patients with single-ventricle physiology often have a poor prognosis and limited therapeutic options. Our data suggest that ARNI may offer some benefit in combination with other oral heart failure medications. However, the significant risk of hypotension in this population especially with shunt dependent physiology, as well as the potential for renal dysfunction, calls for caution in the use of ARNI in single-ventricle patients. The improvement we observed in ventricular function, although promising, must be interpreted with caution, and further studies are needed to confirm these findings.

### Limitations and Future Directions

Our study is limited by its retrospective design, small sample size, and the heterogeneity of the CHD population, which includes patients with a wide variety of congenital defects and varying stages of surgical palliation. While our results suggest that ARNI may hold promise for some patients with CHD, the lack of statistical significance in certain findings such as ventricular function improvements due to the small sample size warrants caution in drawing firm conclusions.

Given these limitations, larger, multicenter, prospective studies are needed to validate our findings and provide more robust data on the safety and efficacy of ARNI in pediatric CHD patients. Future research focusing on identifying which patient subgroups are most likely to benefit from ARNI therapy, particularly those with systemic RV failure or single-ventricle physiology is warranted. In addition, long-term follow-up will be essential to assess whether the short-term improvements in biomarkers, functional status, and ventricular function translate into meaningful clinical outcomes, such as reduced hospitalizations or improvement in transplant free survival.

## Conclusion

In conclusion, sacubitril/valsartan shows potential as a treatment for heart failure in patients with CHD. While we observed improvements in ventricular function in some patients, the high incidences of hypotension and other adverse events highlight the need for careful patient selection and monitoring. Given the unique hemodynamics and complex clinical course of CHD patients, ARNI therapy should be considered on a case-by-case basis, with close attention to potential risks, especially in younger patients and those with single-ventricle physiology. Our study underscores the need for further research into the role of ARNI in patients with CHD. Larger, well-designed trials are essential to establish clear treatment guidelines and optimize the use of ARNI therapy in this vulnerable population.

## Data Availability

No datasets were generated or analysed during the current study.

## Appendix 1: NYHA Functional Classification Definitions

The New York Heart Association (NYHA) functional classification system is used to categorize the severity of symptoms in patients with heart failure:

- **Class I**: No limitation of physical activity. Ordinary physical activity does not cause undue fatigue, palpitation, or dyspnea.
- **Class II**: Slight limitation of physical activity. Comfortable at rest, but ordinary physical activity results in fatigue, palpitation, or dyspnea.
- **Class III**: Marked limitation of physical activity. Comfortable at rest, but less than ordinary activity causes fatigue, palpitation, or dyspnea.
- **Class IV**: Unable to carry out any physical activity without discomfort. Symptoms of heart failure are present even at rest.

## Abbreviations

CHD: Congenital heart disease
HFrEF: Heart failure with reduced ejection fraction
HFpEF: Heart failure with preserved ejection fraction
ARNI: Angiotensin II receptor blocker–neprilysin inhibitor
GDMT: Guideline directed medical therapy
ACC: American College of Cardiology
AHA: American Heart Association
HFSA: Heart Failure Society of America
FDA: Federal Drug Association
NYHA: New York Heart Association
IRB: Institutional review board
ACEi: Angiotensin converting enzyme inhibitor
ARB: Angiotensin II receptor blocker
ACTION: Advanced cardiac therapies improving outcomes network
Kg: Kilogram
mg/kg/dose: Milligrams per kilograms per dose
mg/dose: Milligrams/dose
AKI: Acute kidney injury
BUN: Blood urea nitrogen
eGFR: Estimated glomerular filtration rate
SD: Standard deviation
SGLT2i: Sodium glucose cotransporter 2 inhibitors
PLE: Protein losing enteropathy
HLHS: Hypoplastic left heart syndrome
DORV: Double outlet right ventricle
AVSD: Atrio ventricular septal defect
TOF: Tetralogy of fallot
ccTGA: Congenitally corrected transposition of great arteries
VSD: Ventricular septal defect
PA/VSD: Pulmonary atresia/ventricular septal defect
TAPVR: Total anomalous pulmonary venous return
BSA: Body surface area
BMI: Body mass index
BP: Blood pressure
mmHg: Millimeters of mercury
VAD: Ventricular assist device
MRA: Mineralocorticoid receptor antagonist
PDE5 inhibitor: Phosphodiesterase 5 inhibitor
RV: Right ventricle
LV: Left ventricle
ACHD: Adult congenital heart disease

## References

[1] Bergh N et al (2023) Risk of heart failure in congenital heart disease: a nationwide register-based cohort study. Circulation 147(12):982–984. 10.1161/CIRCULATIONAHA.122.061546 36533451 10.1161/CIRCULATIONAHA.122.061546

[2] Vaikunth SS, Lui GK (2020) Heart failure with reduced and preserved ejection fraction in adult congenital heart disease. Heart Fail Rev 25(4):569–581. 10.1007/s10741-019-09904-z 31873841 10.1007/s10741-019-09904-z

[3] Shaddy R et al (2024) Sacubitril/valsartan in pediatric heart failure (PANORAMA-HF): a randomized, multicenter, double-blind trial. Circulation 150(22):1756–1766. 10.1161/CIRCULATIONAHA.123.066605 39319469 10.1161/CIRCULATIONAHA.123.066605 PMC11593999

[4] Heidenreich PA et al (2022) 2022 AHA/ACC/HFSA guideline for the management of heart failure: a report of the American college of cardiology/American heart association joint committee on clinical practice guidelines. Circulation 145(18):e895–e1032. 10.1161/CIR.0000000000001063 35363499 10.1161/CIR.0000000000001063

[5] Pascual-Figal D et al (2021) Sacubitril-valsartan, clinical benefits and related mechanisms of action in heart failure with reduced ejection fraction. a review. Front Cardiovasc Med 8:754499. 10.3389/fcvm.2021.754499 34859070 10.3389/fcvm.2021.754499 PMC8631913

[6] Cohn JN, Tognoni G, Valsartan I, Heart Failure Trial (2001) A randomized trial of the angiotensin-receptor blocker valsartan in chronic heart failure. N Engl J Med, 345(23):1667–1675. 10.1056/NEJMoa010713 10.1056/NEJMoa010713 11759645

[7] McMurray JJ et al (2014) Angiotensin-neprilysin inhibition versus enalapril in heart failure. N Engl J Med 371(11):993–1004. 10.1056/NEJMoa1409077 25176015 10.1056/NEJMoa1409077

[8] Solomon SD et al (2019) Angiotensin–neprilysin inhibition in heart failure with preserved ejection fraction. N Engl J Med 381(17):1609–1620. 10.1056/NEJMoa1908655 31475794 10.1056/NEJMoa1908655

[9] T. A. C. T. I. O. N. (ACTION) (2025) ACTION medication outpatient titration dosing considerations. https://myactioneducation.org/wp-content/uploads/2023/05/ACTION-Med-Titration-Considerations-20230501.pdf (accessed 26 Mar 2025)

[10] Cohen J (2013) Statistical power analysis for the behavioral sciences

[11] Kemna M, Hong B, Friedland-Little J, Albers E, Law YM (2020) Valsartan/sacubitril in pediatric heart failure. J Heart Lung Transplant. 10.1016/j.healun.2020.01.27532088108

[12] Hale ZE, Prichett L, Jandu S, Ravekes W (2024) Sacubitril-valsartan vs ACE/ARB in pediatric heart failure: a retrospective cohort study. J Heart Lung Transplant 43(5):826–831. 10.1016/j.healun.2024.01.012 38705701 10.1016/j.healun.2024.01.012

[13] Maurer SJ, Pujol Salvador C, Schiele S, Hager A, Ewert P, Tutarel O (2020) Sacubitril/valsartan for heart failure in adults with complex congenital heart disease. Int J Cardiol 300:137–140. 10.1016/j.ijcard.2019.06.031 31242968 10.1016/j.ijcard.2019.06.031

[14] Myhre PL et al (2019) B-type natriuretic peptide during treatment with sacubitril/valsartan: the PARADIGM-HF trial. J Am Coll Cardiol 73(11):1264–1272. 10.1016/j.jacc.2019.01.018 30846338 10.1016/j.jacc.2019.01.018 PMC7955687

[15] Nederend M et al (2023) Tolerability and beneficial effects of sacubitril/valsartan on systemic right ventricular failure. Heart 109(20):1525–1532. 10.1136/heartjnl-2022-322332 37169551 10.1136/heartjnl-2022-322332

[16] Fusco F et al (2023) Safety and efficacy of sacubitril/valsartan in patients with a failing systemic right ventricle: a prospective single-center study. Circ Heart Fail 16(2):e009848. 10.1161/CIRCHEARTFAILURE.122.009848 36458541 10.1161/CIRCHEARTFAILURE.122.009848

[17] Zandstra TE et al (2021) Sacubitril/valsartan in the treatment of systemic right ventricular failure. Heart 107(21):1725–1730. 10.1136/heartjnl-2020-318074 33452121 10.1136/heartjnl-2020-318074 PMC8522462

[18] Appadurai V, Thoreau J, Malpas T, Nicolae M (2020) Sacubitril/valsartan in adult congenital heart disease patients with chronic heart failure—a single centre case series and call for an international registry. Heart Lung Circ 29(1):137–141. 10.1016/j.hlc.2018.12.003 30686641 10.1016/j.hlc.2018.12.003

[19] Lluri G, Lin J, Reardon L, Miner P, Whalen K, Aboulhosn J (2019) Early experience with sacubitril/valsartan in adult patients with congenital heart disease. World J Pediatr Congenit Heart Surg 10(3):292–295. 10.1177/2150135119825599 31084317 10.1177/2150135119825599

[20] Yan L, Loh JK, Tan JL (2021) Sacubitril/valsartan for heart failure in patients with complex adult congenital heart disease—experience from a tertiary centre in Singapore. Int J Cardiol Congenital Heart Disease 6:4. 10.1016/j.ijcchd.2021.100268

