# Correction: Single-Center Experience with Sacubitril/Valsartan in Patients with Congenital Heart Disease

**DOI:** 10.1007/s00246-025-03973-8

**Published:** 2025-07-28

**Authors:** Anusha Konduri, Ashley Duimstra, Ray Lowery, Sunkyung Yu, Ashley Huebschman, Bronwyn Crandall, Tiffany Hunter, Heang M. Lim, Amanda D. McCormick, Kurt R. Schumacher, David M. Peng

**Affiliations:** University of Michigan Congenital Heart Center, Ann Arbor, MI USA

**Correction to: Pediatric Cardiology** 10.1007/s00246-025-03938-x.

In this article, the authors’ names David M. Peng, Heang M. Lim, Amanda D. McCormick and Kurt R. Schumacher were incorrectly written as David Peng, Heang Lim, Amanda McCormick and Kurt Schumacher.

The original article has been corrected.

